# The use of RNA-dependent RNA polymerase for the taxonomic assignment of Picorna-like viruses (order Picornavirales) infecting *Apis mellifera *L. populations

**DOI:** 10.1186/1743-422X-5-10

**Published:** 2008-01-22

**Authors:** Andrea C Baker, Declan C Schroeder

**Affiliations:** 1 Marine Biological Association, The Laboratory, Citadel Hill, Plymouth, PL1 2PB, UK

## Abstract

**Background:**

Single-stranded RNA viruses, infectious to the European honeybee, *Apis mellifera *L. are known to reside at low levels in colonies, with typically no apparent signs of infection observed in the honeybees. Reverse transcription-PCR (RT-PCR) of regions of the RNA-dependent RNA polymerase (RdRp) is often used to diagnose their presence in apiaries and also to classify the type of virus detected.

**Results:**

Analysis of RdRp conserved domains was undertaken on members of the newly defined order, the Picornavirales; focusing in particular on the amino acid residues and motifs known to be conserved. Consensus sequences were compiled using partial and complete honeybee virus sequences published to date. Certain members within the iflaviruses, deformed wing virus (DWV), Kakugo virus (KV) and *Varroa destructor *virus (VDV); and the dicistroviruses, acute bee paralysis virus (ABPV), Israeli paralysis virus (IAPV) and Kashmir bee virus (KBV), shared greater than 98% and 92% homology across the RdRp conserved domains, respectively.

**Conclusion:**

RdRp was validated as a suitable taxonomic marker for the assignment of members of the order Picornavirales, with the potential for use independent of other genetic or phenotypic markers. Despite the current use of the RdRp as a genetic marker for the detection of specific honeybee viruses, we provide overwhelming evidence that care should be taken with the primer set design. We demonstrated that DWV, VDV and KV, or ABPV, IAPV and KBV, respectively are all recent descendents or variants of each other, meaning caution should be applied when assigning presence or absence to any of these viruses when using current RdRp primer sets. Moreover, it is more likely that some primer sets (regardless of what gene is used) are too specific and thus are underestimating the diversity of honeybee viruses.

## Background

Honeybee populations are known to be infected by numerous viruses that reside in colonies yet show no apparent signs of infection [1]. These viruses are often thought to be transmitted by the parasitic mite, *Varroa destructor*, a parasite commonly detected in apiaries [2]. Evidence strongly suggests that when the colony is compromised, for example when infested with *V. destructor*, virus-associated symptoms are observed, including deformed wings and paralysis [2]. Over 18 single-stranded positive sense 'picorna-like' RNA viruses have now been characterised as infectious to the European honeybee, *Apis mellifera *L [1]. Morphologically, these viruses are similar, exhibiting isometric-shaped protein capsids of approximately 30 nm in diameter [3-5]. They also share similarities within their genome sequences, particularly within the helicase, protease and polymerase domains of the replicase polyprotein and also with the order of these 3 domains [6]. The newly defined order Picornavirales, often referred to as the Picorna-like superfamily, encompasses the families Picornaviridae, Dicistroviridae, Comoviridae, Marnaviridae and the Sequiviridae, and the currently unassigned genera, the *Iflavirus*, *Cheravirus*, and *Sadwavirus *[6]. Honeybee viruses of the order Picornavirales include the deformed wing virus (DWV), acute bee paralysis virus (ABPV), Israeli acute paralysis virus (IAPV), chronic bee paralysis virus (CBPV), sacbrood virus (SBV), black queen cell virus (BQCV), Kashmir bee virus (KBV) and the recently identified Kakugo virus (KV). CBPV remains unassigned, while SBV has been classified as a member of the genus *Iflavirus *and BQCV, KBV and ABPV have been assigned to the family Dicistroviridae [7,8]. DWV and KV are considered to also be members of the genus *Iflavirus*, however have not yet been formally classified [9]. In addition to the honeybee viruses, a single-stranded RNA virus replicating within *V. destructor *mites, VDV, has now been identified [10]. The VDV genome has now been sequenced and has been shown to be highly similar to DWV and KV, and is therefore tentatively assigned to the *Iflavirus *genus [10].

The use of RT-PCR to detect the RNA viruses in honeybees is a routinely implemented technique and is often coupled with phylogenetic analyses to investigate similarities or differences between virus isolates. Typically, sequences encoding capsid genes [11,12] and sequences encoding the RNA-dependent RNA polymerase (RdRp) gene [13-16] have been employed for these studies. In particular, the RdRp is considered a good marker for studies concerning RNA virus classification and evolution, with previous research by Koonin & Dolja [17] identifying 8 conserved domains within the RdRp gene of the positive sense single-stranded RNA viruses [6]. The identified domains are considered to have important functions with respect to RNA polymerase activity, with studies involving amino acid substitutions within particular motifs of these domains having significant impacts on the enzymatic activity [18].

In this study, we assessed the suitability of the RdRp to not only detect, but to differentiate between the different picorna-like viruses found within the order Picornavirales. This is considered especially important in light of the ever increasing entries in sequence databases of viruses belonging to the order Picornavirales and the tentative assignments of viruses to particular families/genera, often based on partial sequences [19,20]. We also analyse the validity of using the RdRp as a marker for studying viruses infecting honeybees.

## Results

### Analysis of RdRp conserved domains across the order Picornavirales

The recently defined order Picornavirales has 8 members [6] and closer analysis of the conserved domains identified by Koonin and Dolja [17] based on a multiple sequence alignment of 46 virus sequences was undertaken (Table 1). Within domain I of the order Picornavirales the Lysine (K) and Aspartic acid (D) residues in the 4^th ^and 5^th ^positions are conserved across all members; the family Dicistroviridae and the genus *Iflavirus *are the most variable in this domain, with only 3 and 2 conserved amino acids respectively, and these two members were the only two not to have the conserved motif KDE. Domain II was highly variable, where only one amino acid, Arginine (R), was conserved for 7 out of the 8 members, the exception being the family Dicistroviridae, which had a potential Lysine (K) substitution at this position for BQCV, Triatoma virus (TRV) and Himetobi P virus (HiPV), yet both have basic amino acid properties (Table 2). In addition, the family Picornaviridae have an insertion in this domain that was absent in all the other members. In domain III a deletion and a substitution of the otherwise conserved amino acid Tryptophan (W) separated the family Picornaviridae from the others. The amino acid Glycine (G) was nonetheless found to be conserved amongst all of the members. With the exception of the genus *Ilfavirus*, all members of the order Picornavirales have 2 aspartic acid (D) residues and 2 conserved sites of amino acids with aromatic side chains in domain IV. The genus *Iflavirus *had a substitution of either Glycine (G) or Serine (S) at the 2^nd ^conserved aspartate site (Table 2). Domain V is the most conserved domain with the consensus sequences PSGxxxTxxxN occurring in 5 out of 8 members. All the 8 members possess the GDD motif in domain VI, while YGDD (in domain VI) and FLKR motif (in domain VII) were conserved in 87.5% and 75% of the members, respectively. Domain VIII was the least conserved with the *Sadwavirus, Cheravirus*, Sequiviridae and Marnaviridae having the shared PLxxxxI motif.

**Table 1:** Virus sequences used to create consensus sequences of the RdRp for the families/genera comprising the Picornavirales.

Virus	Abbreviation	Family/Genus	Accession Number
Equine rhinitis B virus 1	ERBV-1	Picornaviridae	NP_740368
Encephalomyocarditis virus	EMCV	Picornaviridae	NP_056777
Theilers encephalomyelitis virus	TMEV	Picornaviridae	AAA47928
Foot and mouth disease virus	FMDV	Picornaviridae	CAA25419
Equine rhinitis A virus	ERAV	Picornaviridae	NP_740383
Porcine teschovirus 1	PTV-1	Picornaviridae	CAB40546
Porcine teschovirus 8	PTV-8	Picornaviridae	AAK12387
Aichi virus	AiV	Picornaviridae	NC_001918
Bovine kobuvirus	BKV	Picornaviridae	NC_004421
Poliovirus 1	PV1	Picornaviridae	P03300
Bovine enterovirus	BEV-1	Picornaviridae	AAZ73355
Human rhinovirus 89	HRV-89	Picornaviridae	P07210
Human hepatitis A virus	HAV	Picornaviridae	P08617
Simian hepatitis A virus	SHAV	Picornaviridae	CAA33490
Human parechovirus 3	HPeV-3	Picornaviridae	CAI64373
Ljungan virus	LV	Picornaviridae	NP_705884
Cowpea severe mosaic virus	CPSMV	Comoviridae	NP_734062
Red clover mottle virus 2	RCMV-2	Comoviridae	P35930
Broad bean wilt virus 2	BBWV-2	Comoviridae	AAX12875
Tobacco ringspot virus	TRSV	Comoviridae	Q6UR06
Beet ringspot virus	BRV	Comoviridae	P18522
Satsuma dwarf virus	SDV	*Sadwavirus*	NP_734025
Strawberry mottle virus	SMoV	*Sadwavirus*	NP_733954
Apple latent spherical virus	ALSV	*Cheravirus*	NP_734022
Cherry rasp leaf virus	CRLV	*Cheravirus*	YP_081454
Parsnip yellow fleck virus	PYFV	Sequiviridae	BAA03151
Rice tungro spherical virus	RTSV	Sequiviridae	AAA66056
Kashmir bee virus	KBV	Dicistroviridae	AAG28568
Acute bee paralysis virus	ABPV	Dicistroviridae	AAN63804
Taura syndrome virus	TSV	Dicistroviridae	ABB17263
Cricket paralysis virus	CrPV	Dicistroviridae	AAF80998
Drosophila C virus	DCV	Dicistroviridae	AAC58807
Black queen cell virus	BQCV	Dicistroviridae	AAF72337
Triatoma virus	TrV	Dicistroviridae	AAF00472
Himetobi P virus	HiPV	Dicistroviridae	BAA32553
Plautia stali intestine virus	PSIV	Dicistroviridae	EAA21898
Aphid lethal paralysis	ALPV	Dicistroviridae	AAN61470
Rhopalosiphum padi virus	RhPV	Dicistroviridae	AAC95509
Deformed wing virus	DWV	*Iflavirus*	CAD34006
Kakugo virus	KV	*Iflavirus*	YP_015696
Varroa destructor virus	VDV	*Iflavirus*	YP_145791
Sacbrood virus	SBV	*Iflavirus*	AAD20260
Venturia canescens picornalike virus	VcPLV	*Iflavirus*	AA537668
Infectious flacherie virus	IFV	*Iflavirus*	BAA25371
Perina nuda virus	PnV	*Iflavirus*	AAL06289
Heterosigma akashiwo virus	HaRNAV	Marnaviridae	NP_944776

**Table 2:** Consensus sequences of the RdRp for members of the Picornavirales for the domains identified by Koonin & Dolja [17]. Conserved amino acids are highlighted and any conserved amino acid properties.

Virus Family/Genus	Domain I	Domain II	Domain III	Domain IV	Domain V	Domain VI	Domain VII	Domain VIII
Picornaviridae	XXX**KD**ELRXXX	XXXXXXXXXXXXXRXXXXXXXXXXXXXXX	X**G**XXP-XXXXXX	X**D**XXXXDXXXX	XGXXPS**G**XXX**T**XXXNXXXNXXXXXXXX	XXXY**GDD**XXX	XXXXFLKRX	XXXXXXXXXX
Comoviridae	EXX**KD**EXLXXR	XFXXLXXXXNXXXRXXFLXXXXXXX-XXR	V**G**XXXXXXEWXX	C**D**YXXFDGXXX	XXGIXX**G**XXL**T**VXXNSXXNEXLXXXXX	XXXY**GDD**NLI	XXXDFLKRX	XXXXXXXXXX
Sadwavirus	ACA**KD**EKTXXR	IFEILPFXXNIXXRXYXXFXMQXXM-XXH	V**G**XNVYSXSWDX	G**D**YXGFDTXTP	XGGTPS**G**FAX**T**VXINSVVNXFYLXWXW	XSXY**GDD**NXV	XEXDFLKRX	PLXKXXIEER
*Cheravirus*	DFP**KD**EKTXXK	LFXILPVDYNILVRKYFLSFVSXXM-XXH	V**G**IDXXSNEWSI	G**D**YSRFDGITP	TSGIPS**G**FPL**T**VIVNSLVNXFFXHFXY	YAXY**GDD**NLX	EKVDFLKRX	PLNXVNITER
Sequiviridae	ECX**KD**ERRXLX	XFXILXXEXNXXXRXXFXDFXXXVM-XXR	V**G**INPXSXEWSD	G**D**XXXFDGXXX	XXGXPS**G**FXM**T**VIFNSFXNXXXXXXAW	XXXY**GDD**NXV	XXXXFLKRX	PLXKXSIEEX
Dicistroviridae	XXL**KD**XXXXXX	XFXXXXXXXXXXXYXXXXXXXXXXX-XXX	X**G**XNXXSXXWXX	G**D**XXXXDXXXX	XXXXPS**G**XXX**T**XXXNXXXXXXXXXXXX	XXXY**GDD**XXX	XXXXXXKRX	PXXXXXXXXX
*Iflavirus*	XXX**KD**XXXXXX	XXXXXPXXXXXXXRXXXXXFXXXXX-XXX	XGXXXXXXXWXX	X**D**YXXXXXXXX	XXGXXX**G**XXX**T**XXXNXXXNXXXXXXXX	XXXX**GDD**XXX	XXXXXLXXX	XXXXXXXXXX
Marnaviridae	ATK**KD**EARLIG	TFYAASMNVIMAVRKYFCPVLQALK-ANP	I**G**TNAFGKDWAD	G**D**YSSFDMSHN	IGWVMS**G**VPL**T**AELSSTLNQIYMRVVW	LIVY**GDD**NNA	EDAEFLKRL	PLSWDSINKR
Properties		2 2 3 2 2	1 4	4 4	1 55		413	2

X: variable position within family/genus

-: deletion

1: Aliphatic amino acid

2: Hydrophobic amino acid

3: Basic amino acid

4: Aromatic amino acid

5: Neutral amino acid

Bold type and underline type indicating 100 and > 75% amino acid conservation respectively.

### Analysis of RdRp conserved domains amongst the honeybee viruses

With the exception of CBPV (which remains unassigned), the honeybee viruses analysed in this study have been assigned or tentatively assigned (these will be discussed as assigned viruses for the purpose of this paper) to 2 separate groups within the order Picornavirales, the family Dicistroviridae and the genus *Iflavirus*. Analysis of the consensus sequences for these 3 main groupings across all 8 domains was undertaken on 139 virus sequences (Table 3), and showed conserved amino acids present in the family Dicistroviridae that are absent in the genus *Iflavirus*, and vice versa (Table 4). CBPV, which has only had the RdRp gene partially sequenced, is distinct to the others, sharing little similarity, with the exception of 4 amino acids in domain V and the GDD motif in domain VI.

**Table 3:** Virus sequences used to create consensus sequences for the RdRp of Honeybee viruses of the Picornavirales.

Kashmir bee virus	KBV	Dicistroviridae	AAG28568 AAG28567 AAG28569 AAG28570 AAG28571 NP_851403 AAP32283 AAK13621 AAK13620 AAK13619 AAV52628 AAG33697 AAG33696 AAG33695 AAG33694
Acute bee paralysis virus	ABPV	Dicistroviridae	AAG13118 AAN63803 AAN63804 DQ434968–DQ434990

Israeli acute paralysis virus	IAPV	Dicistroviridae	YP_001040002 AAV6479

Black queen cell virus	BQCV	Dicistroviridae	AAF72337 AAU10095 AAU10094 DQ434991

Sacbrood virus	SBV	*Iflavirus*	AAL79021 AAD20260 AAU10097 DQ434992

Deformed wing virus	DWV	*Iflavirus*	CAD34006 AAP49008 AAP49283 DQ434893–DQ434967

Kakugo virus	KV	*Iflavirus*	YP_015696

Varroa destructor virus	VDV	*Iflavirus*	YP_145791

Chronic bee paralysis virus	CBPV	Unassigned	AAM46093 AAM47564 AAM47565 AAM47566 AAM47567 AAM47568 AAM47569 AAM47570 AAM47571

**Table 4:** Compilation of consensus sequences of the RdRp for the Picornavirales Honeybee viruses sequenced to date for the domains identified by Koonin & Dolja [17].

Virus	Family/Genus	Domain I	Domain II	Domain III	Domain IV	Domain V	Domain VI	Domain VII	Domain VIII
KBV	Dicistroviridae	**D**T**LKD**ERRPIE**K**	V**F**SNG**P**MDFSIAFRM**Y**YLGFI**A**HLMENR	I**G**TN**V**Y**S**QD**W**SK	G**D**FST**F**DGSLN	THSQP**SG**NPA**T**TPL**N**CFINSMGLRMCFSI	LVSY**GDD**NVI	QDVQY**LK**RK	P**L**CMDTILEM
ABPV	Dicistroviridae	**D**T**LKD**ERRPIE**K**	V**F**SNG**P**MDFSITFRM**Y**YLGFI**A**HLMENR	I**G**TN**V**Y**S**QD**W**HK	G**D**FST**F**DGSLN	THSQP**SG**NPA**T**TPL**N**CFINSMGLRMVFEL	IVSY**GDD**NVI	EDVQY**LK**RK	P**L**SMDTILEM
IAPV	Dicistroviridae	**D**T**LKD**ERRPIE**K**	V**F**SNG**P**MDFSIAFRM**Y**YLGFI**A**HLMENR	I**G**TN**V**Y**S**GD**W**SK	G**D**FST**F**DGSLN	THSQP**SG**NPA**T**TPL**N**CFINSMGLRMCFAI	MVSY**GDD**NVI	KDVQY**LK**RK	P**L**CMDTILEM
BQCV	Dicistroviridae	**D**T**LKD**ERKPKH**K**	M**F**SNG**P**IDYLVWSKM**Y**FNPIV**A**VLSELK	V**G**SN**V**Y**S**TD**W**DV	G**D**FEG**F**DASEQ	CKSLP**SG**HYL**T**AII**N**SVFVNLVMCLVFME	IVAY**GDD**HVV	EDVSY**LK**RN	P**L**SLDVVLEM
SBV	*Iflavirus*	**D**T**LKD**ERKLPE**K**	V**F**CNP**P**IDYIVSMRQ**Y**YMHFV**A**AFMEQR	V**G**IN**V**Q**S**TE**W**TL	I**D**YSN**F**GPGFN	KCGSP**SG**API**T**VVI**N**TLVNILYIFVAWET	LFCY**GDD**LIM	LNSTF**LK**HG	A**L**AWSSINDT
DWV	*Iflavirus*	**D**C**LKD**TCLPVE**K**	I**F**SIS**P**VQFTIPFRQ**Y**YLDFM**A**SYRAAR	I**G**ID**V**N**S**LE**W**TN	G**D**YKN**F**GPGLD	PCGIP**SG**SPI**T**DIL**N**TISNCLLIRLAWLG	LVCY**GDD**LIM	QTATF**LK**HG	N**L**DKVSVEGT
VDV	*Iflavirus*	**D**C**LKD**TCLPVE**K**	I**F**SIS**P**VQFTIPFRQ**Y**YLDFM**A**SYRAAR	I**G**ID**V**N**S**LE**W**TN	G**D**YKN**F**GPGLD	PCGIP**SG**SPI**T**DIL**N**TISNCLLIRLAWQG	LVCY**GDD**LIM	QTATF**LK**HG	N**L**DKVSIEGT
KV	*Iflavirus*	**D**C**LKD**TCLPVE**K**	I**F**SIS**P**VQFTIPFRQ**Y**YLDFM**A**SYRAAR	I**G**ID**V**N**S**LE**W**TN	G**D**YKN**F**GPGLD	PCGIP**SG**SPI**T**DIL**N**TISNCLLIRLAWLG	LVCY**GDD**LIM	QTATF**LK**HG	N**L**DKVSVEGT
CBPV	Unassigned					EGTRC**SG**DPH**T**SIG**N**GFINAFIIWLCLRK	SAHE**GDD**GIV		

Bold type and underline type indicating 100 and > 75% amino acid conservation for domains I-IV, VII-VIII respectively.

Bold type and underline type indicating 100 and > 77.8% amino acid conservation for domains I-IV & VII-VIII respectively.

### Family Dicistroviridae

In general, BQCV shared more conserved motifs with other members within the family Dicistroviridae, but it also had the most amino acid substitutions across all domains (Table 4). The amino acid sequences of both domains I and IV are identical in the 3 viruses, KBV, IAPV and ABPV, yet changes were noted at the nucleotide level (data not shown). Within domain II, KBV, ABPV and IAPV are identical except for 1 amino acid substitution in ABPV, where Alanine (A) is substituted for Threonine (T) (Table 4). The end of domain V and the start of domain VI show the greatest region of amino acid variability in these 3 viruses, with each of the viruses having 2 unique amino acid residues each (Table 4). At the nucleotide level, ABPV differed to KBV and IAPV, and within the ABPV sequences analysed, domain II was least conserved with 8 nucleotide substitutions, whereas no substitutions were detected in domain I, 0 in domain III and 3 in domain IV (data not shown).

### Genus *Iflavirus*

SBV shows the most amino acid differences in this group, with DWV, VDV and KV showing a high level of similarity. These 3 viruses are identical at the amino acid level in domains I, II, III, VI and VII (Table 4). Only 2 amino acid substitutions are evident in VDV, in domains V and VII, where Glutamine (Q) is substituted for Lysine (L) and Isoleucine (I) is substituted for Valine (V) respectively. Nucleotide substitutions are, however, detected in all 8 domains both within the DWV sequences and also with the KV and VDV sequences. VDV was different from the two identical nucleotide sequences of KV and DWV by 1 nucleotide substitution in domain I (data not shown). Domain II was more variable for DWV with nucleotide substitutions at 8 sites (35 isolates were analysed), and 4 within KV and 11 with VDV (data not shown).

Overall, domains I & II were the most conserved amongst all the honeybee viruses analysed and thus the boundary that separated the members of the family Dicistroviridae and genus *Iflavirus *was less clear. Domains III to VIII revealed clearer separation between these two members (Table 4). In fact the conservation of amino acids within domains V and VI is in agreement with CBPV belonging to a different genus if not family.

The consensus sequences for the 8 domains of the honey bee viruses were force joined to form a contiguous sequence and were aligned against each other to compare the sequences (Table 5). The iflaviruses, DWV, VDV and KV share greater than 98% sequence identity, with KV and DWV being identical, however, shared only 51% and 52% homology with the other iflavirus, SBV. Similarities between the aforementioned iflaviruses and the dicistroviruses, ABPV, IAPV, KBV and BQCV, were less than 43%. Within the dicistroviruses, IAPV and KBV shared the highest sequence similarity of 96%, with IAPV and ABPV sharing 92% similarity and KBV and ABPV sharing 93%. Similarities of these 3 viruses with BQCV were considerably lower, ranging from 47–51 % (Table 5).

**Table 5:** Percentage homology between the honeybee viruses described in this study, acquired by force joining domains I-VIII of the RdRp and conducting pairwise comparisons using BLAST.

	ABPV	IAPV	BQCV	SBV	DWV	VDV	KV
KBV	93	96	47	39	43	43	43
	ABPV	92	51	39	41	41	41
		IAPV	47	38	41	41	41
			BQCV	37	30	30	30
				SBV	51	52	51
					DWV	98	100
						VDV	98

## Discussion

### Validation of RdRp as a genetic marker for the order Picornavirales

The order Picornavirales share a common virion structure, single-stranded positive sense RNA genome, 3' poly A tail and a 5' VPg [6]. The viruses of this order encode a type I RdRp domain within the replicase polyprotein that exhibits 8 conserved motifs [17]. Comparative analysis of the RdRp (Table 2) revealed that certain amino acid residues or motifs are conserved amongst all of the domains of this order, with the yGDDn motif located in domain VI seemingly the most conserved. In addition, it is common where an amino acid is substituted in a particular group for it to retain similar properties to the substituted amino acid. The FLKR motif in domain VII is one such example, with the Phenylalanine (F) in the family Dicistroviridae and genus *Iflavirus *often being substituted to Tyrosine (Y), which shares the property of being an aromatic amino acid. Hence, the comparison of the consensus amino acid sequence for each group supports the current classification of these viruses together within this order and suggests that their RdRp share similar properties or activities (Table 2). The highly conserved GDD motif is thought to have an imperative role in RdRp activity, with the 1st aspartate residue in the motif being shown to be involved in the coordination of magnesium ions during nucleotidyltransfer catalysis [21]. If this amino acid is substituted, viral replication and RNA synthesis has been shown to cease [18].

The analysis of the RdRp of the order Picornavirales shows that there is enough sequence variability for the subdivision of this order into the 8 families and genera, as previously assigned based on features described by Christian et al. [6] (Table 2). Briefly, these characteristic features include the conserved order of core non-structural protein domains, a polyprotein gene expression strategy processed exclusively by virus proteinases, a pseudo-T3 isocahedral symmetry of capsids, a 3–4 kDa VPg with few characteristic features, a hydrophobic domain between the helicase and VPg, a 3C-like Cysteine proteinase, a type II helicase domain and type I polymerase domain [6]. Unique amino acids or motifs can be identified in the RdRp of particular families or genera, meaning that they can be differentiated. For example, the genus *Sequivirus *has a conserved KDERR motif in domain I, whereas the genus *Cheravirus *has a KDEKT motif (Table 2). The families Picornaviridae, Dicistroviridae and genus *Iflavirus *show the highest degree of variability and could potentially be subdivided further within their respective group as there appears to be obvious subdivisions that could be applied (data not shown). One potential subdivision could be within the family Dicistroviridae, with KBV, ABPV, CrPV, TSV and DCV forming a genus due to their high similarity within this family. Future analyses could address whether these viruses differ in any other way to the other members of the family Dicistroviridae in their RdRp enzymology or with respect to their epidemiology, transmission or persistence. Much more information is being brought to light regarding the importance of the motifs in the structure and functioning of RdRp [22]. As RdRp is universal in the positive sense RNA viruses it makes it a key focus for the understanding of viral replication, evolution and pathogenesis. Further structural and biochemical studies will provide more clues regarding RdRp, which, based on these alignments, can be tentatively predicted in all other viruses sharing these motifs.

### Validation of RdRp for the differentiation of honeybee viruses

With the RdRp being confirmed as a good marker for resolving hierarchical structures within the order Picornavirales, sequences of honeybee viruses deposited in GenBank were investigated further to assess the application of RdRp for differentiating between these viruses. Within the family Dicistroviridae, BQCV shows consistent amino acid differences with KBV, IAPV and ABPV across all 8 domains, yet is more closely related to these viruses than any other honeybee virus (Table 4). KBV, IAPV and ABPV, however, are much more similar, being identical at the amino acid level in domains I and IV (Table 4). KBV and IAPV are the most similar, sharing 96% amino acid sequence identity (Table 5). The amino acid differences between these three viruses are not at key conserved sites which are considered to be important in RdRp structure and function. This high amino acid similarity is also mirrored (at a lesser extent) in the nucleotide sequences, with de Miranda et al. [7] reporting a 70% nucleotide identity between ABPV and KBV. Serologically and biologically, KBV, IAPV and ABPV are very similar, with BQCV being the more different in this family [8], and this is also reflected in the RdRp gene. The symptoms associated with BQCV are not observed in association with any of the other dicistroviruses, with the queen brood being seen to darken and die, the queen cell walls turning black, and being additionally known to be transmitted by the parasite, *Nosema apis *[5]. ABPV, IAPV and KBV have less easily defined symptoms, such as trembling, crawling bees, or indeed no overt symptoms at all, making them difficult to diagnose in the field. Sequence analysis of the RdRp suggests they are highly related and it is possible that they diverged very recently and should be considered as variants of each other.

The RdRp lacks a proof reading function and hence is more prone to errors, leading to frequent nucleotide changes and subsequently, amino acid substitutions [23,24]. The amino acid sequence is the important factor in the functionality of this enzyme playing pivotal roles in maintaining the integral conformation, and coordinating the discrimination of sugars and coordinating ions. The conserved motifs observed within these honeybee viruses are obviously important in the RdRp activity, otherwise their persistence within the RdRp would have not have occurred. Nucleotide substitutions within this gene have transpired [25] yet have not translated into significant changes in the amino acid composition, implying the core functionality has remained the same for ABPV, IAPV and KBV. IAPV has recently been implicated as responsible for colony collapse disorder (CCD), where colonies, particularly in America, have been seen to suddenly die without any detection of virus-like symptoms [26]. Here we propose that IAPV is also a variant of the ABPV and KBV, having evolved as a more aggressive pathogen. Certainly, there are divergent regions of sequences present within the genomes of these viruses, with de Miranda et al. [7] describing regions of only 33% homology between ABPV and KBV, such as regions between the helicase and 3C-protease domains and the non-structural polyprotein. RNA-based viral genomes are more likely to mutate due to the error prone nature of RdRp, however certain regions do not have a strong selection pressure to retain a sequence, which is why these regions are more likely to be variable. Subsequently, these regions are less appropriate when used solely for inferring virus taxonomy.

At this point it is also important to re-evaluate the data obtained from the particular primer sets employed in RT-PCR for the routine detection of the viruses in colonies. Analysis of primers employed by Tentcheva et al. [16] and Baker & Schroeder [25], for the detection of ABPV suggests that they may have also amplified IAPV. Only 4 out of 21 nucleotides (mainly at the 5' end of the oligonucleotide) in the forward primer were different to the IAPV sequence, and only 2 out of 20 differed in the reverse primer. Due to the imprecise nature in preparing PCRs, i.e. different reagents, quality of samples, different thermocyclers etc., and even when stringent PCR conditions are used, the detection of IAPV with this primer set cannot be discounted. Hence, when interpreting results on the occurrence and distribution of these viruses care must be taken as functional variants may either be amplified or missed. Sequencing negates this problem, to an extent, however, it would need to be performed on every sample analysed to confirm the exact variant detected. Other studies have utilised the structural polyprotein for the confirmation of presence or absence of honeybee viruses in colonies [11,27], however, depending on the purpose of the study it may actually be more appropriate to design primers within the RdRp gene, ensuring most, if not all variants, are captured.

A similar scenario was detected in the genus *Iflavirus *with VDV, KV and DWV sharing a greater than 98 % homology across the 8 domains and only 2 amino acid substitutions (Tables 4 &5). Again in this genus, a lower homology was identified with the other member of the group, SBV, with 51/52% homology, confirming their division as separate virus 'species' (Table 5). As with BQCV, in the family Dicistroviridae, SBV is very different in observed symptoms in comparison to the symptoms seen in the other *Apis mellifera *infecting iflaviruses, supporting the suggestion that it may be more divergent. The implications of the strong homology and amino acid conservation amongst the iflaviruses, VDV, KV and DWV, are that they are highly similar and most likely have similar replication efficiencies. Consequently, we propose these viruses share a recent common ancestor. Certainly this concept has already been proposed by Lanzi et al. [9] where, unlike in ABPV and KBV [7], none of these potential variants show geographical distinction, and the phylogenetic analysis of the RdRp shows no divisions that correlate to different regions [9]. Our results are consistent with those of a recent study on DWV strains detected across the world, where a low nucleotide sequence divergence is also observed in the helicase and structural genes of this virus [28]. No clear geographical pattern of distribution was identified based on the phylogenetic analysis of these genes either, suggesting that other genes within these viruses are also highly conserved. In this study by Berenyi et al. [28], DWV was indeed separated into a separate clade from VDV and KV, yet this grouping was supported by bootstrap values of less that 70, questioning the robustness of this separation. We therefore support the variant hypothesis of Lanzi et al. [9] as other observations, such as both VDV and DWV replicating within the *Varroa *mite (KV has not yet been tested) [10], also lead to the same conclusion. However, differences arise when addressing the symptoms involved with these virus infections, with KV and DWV manifesting different symptoms within the honeybees. KV has been show to cause aggressiveness in the bees [29], being localised in the brain tissue, and with DWV causing deformed, crumpled wings and not being localised to specific body part [30]. The pathological effect VDV has on the mites and also the honeybees has yet to be deciphered, however, from genomic analysis by Ongus et al [10], VDV has been confirmed as being highly similar to DWV and KV, having an 84% sequence identity. It is suggested that variations existing in other parts of the genomes of these viruses have contributed to their pathological characteristics, for example the specificity of KV to brain tissues, and the ability of DWV and VDV to replicate in mites. This virus may have nucleotide changes in the structural polyprotein that have transpired to amino acid changes and consequently induced an alteration of host tissue recognition. Indeed, this has been observed in the canine paravirus (CPV), a virus infectious to cats, minks, racoons and dogs, yet the ancestor virus, feline panleukopenia virus (FPV), cannot infect dogs. It was resolved that 2 amino acid residue changes in the capsid protein of FPV, resulted in the expansion of this virus host range, creating the CPV variant, hence it is feasible that a similar scenario may have emerged in the honeybee viruses [31].

In addition, the detection of these iflaviruses through RT-PCR can be unreliable, depending on the purpose of the study, as the likelihood of detecting all the known variants is high. DWV-specific primers used by Tentcheva et al. [16] and Baker & Schroeder [25] had only 1 mismatch in the forward primer with KV and no mismatches in the reverse; therefore it is plausible that this variant was also detected. A recent study by Chen et al. [14] also highlights this aspect when they used quantitative PCR to investigate DWV prevalence, with the forward primer containing no mismatches for KV and 1 for VDV, the reverse having no mismatches for KV and 2 mismatches for VDV, and the probe have 0 mismatches for KV and 1 for VDV respectively. Thus, this should be considered when interpreting their results, as it is possible that they were detecting different or even missing other variants in different tissues and/or bee types.

To date, only a region of the RdRp of CBPV has been sequenced and based on traditional classification requirements, it is difficult to assign a family/genus for this virus. Based on our analysis CBPV is clearly a member of the order Picornavirales, however, it appears that it is very divergent from the other characterised honeybee viruses and thus should be assigned as the type strain for a new genus and/or family.

## Conclusion

We have validated the use of the RdRp as a taxonomic marker for the classification of the order Picornavirales and, to an extent, for the viruses infecting the honeybee. The evidence supports the assignment of DWV, VDV and KV as variants of the same virus, with it also being proposed that ABPV, IAPV and KBV, are also variants of the same virus. We suggest that care should be taken when using molecular tools to ascertain whether certain viruses are present in any given sample and thus will affect the prediction of cause and effect. The data presented here provides further foundations for understanding the ecology of these viruses and the interactions they have with their hosts, therefore being useful for beekeeping practises. The results potentially also provide further information on the evolution of these honeybee viruses in the context of the order Picornavirales.

## Methods

### Validation of RdRp oligonucleotide probes

Multiple amino acid and nucleotide sequences of the RNA-dependent RNA polymerase (RdRp) protein for the single-stranded RNA viruses were selected from NCBI (Tables 1 &3) and were aligned using ClustalW using the default settings [32]. Conserved regions spanning motifs I to VIII of the RdRp, as defined by Koonin & Dolja [17], were used for analysing the suitability of this gene as a marker. Published oligonucleotides were analysed against this alignment to assess suitability to differentiate between inter- and intra-species variations within the Picornavirales

## Competing interests

The author(s) declare that they have no competing interests.

## Authors' contributions

ACB performed all the experimental work, carried out the genetic analysis and wrote the manuscript. DCS co-ordinated the development of the project, performed the multiple sequence alignments and oversaw the research.
